# Adaption and validation of the Mississippi Aphasia Screening Test to Estonian speakers with aphasia

**DOI:** 10.1002/brb3.1188

**Published:** 2018-12-19

**Authors:** Aaro Nursi, Marika Padrik, Liisa Nursi, Maarja Pähkel, Liis Virkunen, Anne Küttim‐Rips, Pille Taba

**Affiliations:** ^1^ Department of Neurology and Neurosurgery University of Tartu Tartu Estonia; ^2^ Department of Neurology Tartu University Hospital Tartu Estonia; ^3^ Institute of Education University of Tartu Tartu Estonia

**Keywords:** aphasia, Estonia, Mississippi Aphasia Screening Test, stroke

## Abstract

**Objectives:**

The Mississippi Aphasia Screening Test (MAST) is a brief screening tool for assessing the expressive and receptive language abilities of patients with aphasia. The goal of this study was to adapt and validate the MAST into the Estonian language. The discriminant validity and internal consistency of the test were examined, as well as its sensitivity and specificity.

**Methods:**

The MASTest was administered in 50 left hemisphere stroke patients with aphasia (LHA+ group) in the acute phase after the stroke and 126 healthy volunteers in a control group (CG), stratified by age and level of education. Nonparametric tests were used to get normative values, compare the values of the MASTest scores between the LHA+ group and the CG, and to assess the discriminant validity, internal consistency, sensitivity, and specificity of the MASTest.

**Results:**

The summary scores: total score (MASTest‐T), expressive score (MASTest‐E), and receptive score (MASTest‐R) correlated with age and educational level, and the normative values were adjusted accordingly. The LHA+ group showed more impairment than the CG in all subtests and summary scores. The internal reliability of the MASTest was high for the whole sample and LHA+ group. The sensitivity and specificity of the MASTest using the 5th percentile were 74% and 94%, respectively, but using receiver operating characteristic (ROC) analysis, it was 89% and 80%.

**Conclusion:**

The MASTest is a valid screening tool for evaluating expressive and receptive language abilities in Estonian patients with aphasia in early stroke. The MASTest is the first validated aphasia screening test for Estonian‐speaking people, who number less than one million worldwide.

## 1 INTRODUCTION

Aphasia is one of the most common and debilitating consequences of both the acute and chronic phases of stroke. Aphasia is present in 21%–38% of patients with acute stroke (Berthier, 2005; Laska, Hellblom, Murray, Kahan, & Arbin, 2001; Pedersen, Jorgensen, Nakayama, Raaschou, & Olsen, 1995), severely affecting patients’ ability to communicate, and therefore has a negative impact on quality of life (Hilari, 2011; Spaccavento et al., 2014). Early identification and diagnosis of aphasia is an essential step toward maximizing therapy gains and improving language recovery outcomes (Salter, Jutai, Foley, Hellings, & Teasell, 2006).

Lengthy aphasia test batteries may be burdensome for stroke patients in poor health or with severe aphasia. Instead, it is reasonable to use considerably shorter aphasia screening tests in the acute phase of stroke (Al‐Khawaja, Wade, & Collin, 1996). There are several aphasia screening methods reported in the literature (Hachioui et al., 2017; Salter et al., 2006) that help with bedside evaluation to identify aphasia with the purpose of early diagnosis, therapy selection, and an improved prognosis. In this study, the MAST was chosen because it does not burden the patient and provides a broad overview of language abilities.

The MAST has become a widely used screening tool for identifying stroke patients with aphasia. The original English version of the MAST was published in 2002 (Nakase‐Thompson et al., 2002) and validated in 2005 (Nakase‐Thompson et al., 2005). The test has since been validated in the Czech, Spanish, Telugu, and Persian languages, but no Estonian‐language version yet exists (Khatoonabadi, Nakhostin‐Ansari, Piran, & Tahmasian, 2015; Kostalova et al., 2008; Nagendar & Ravindra, 2012; Romero et al., 2012).

The MAST was developed as a brief, repeatable screening measure for individuals with severely impaired language skills. It was designed for a dynamic assessment of changes in language abilities over time and requires 5–10 min to administer (Nakase‐Thompson et al., 2005).

Estonia participates in international stroke studies including patients with aphasia (Budincevic et al., 2015; Kõrv et al., 2014). However, standardized screening tests for aphasia are still lacking for medical personnel in Estonia. Once validated, the MASTest will be able to provide more reliable data for these studies.

The aim of the present study was to linguistically and culturally adapt the MAST into the Estonian language. The discriminant validity and internal consistency of the test were examined as well as its sensitivity and specificity.

## 2 METHODS

### 2.1 Participants

Nonconsecutive first‐time stroke patients with ischemic or hemorrhagic unilateral left hemisphere stroke (documented by neurological examination and computed tomography) admitted to the Department of Neurology of the Tartu University Hospital between 1 January 2014 and 31 April 2015 underwent nonstandard logopedic examination, and those with documented aphasia were recruited.

Persons with recurrent episodes of stroke, severe impairment of sight or hearing with no adequate correction available, or prestroke dementia diagnosis in previous medical history, and those with ischemic or hemorrhagic unilateral left hemisphere stroke without aphasia were excluded.

The CG was recruited during the same testing period as stroke patients from healthy volunteers who agreed to cooperate, who had no known or suspected speech or language impairment, and who spoke Estonian as their native language. Subjects with a history of neurological dysfunction, such as stroke, traumatic brain injury, dementia, and severe impairment of sight or hearing with no adequate correction available, were excluded.

Altogether, 50 subjects with documented aphasia who complied with the inclusion criteria, among them 20 men and 30 women, with a median age of 72.5 (range 55–90, QD = 13.75) years, were included in the LHA+ group. There were 25 patients with a basic level of education, 21 with a secondary level of education, and 4 with a high level of education (Table 1).

**Table 1:** Sociodemographic variables

Variable	LHA+ group (*n* = 50)	CG (*n = *126)	CG1 (*n = *63)	Level of significance (LHA+ group vs. CG1)
Age (years) (median and range)	72.5 (55–90)	54.5 (18–89)	72 (55–89)	*p* > 0.05
Sex (males)	20 (40.0%)	63 (50.0%)	30 (47.6%)	*p* > 0.05
Handedness (% right handed)	50 (100%)	126 (100%)	63 (100%)	*p* > 0.05
Level of education: Basic	25 (50.0%)	42 (33.3%)	22 (34.9%)	*p* > 0.05
Secondary	21 (42.0%)	42 (33.3%)	21 (33.3%)	*p* > 0.05
High	4 (8.0%)	42 (33.3%)	20 (31.7%)	*p* < 0.01**

Note

LHA+ group: left hemisphere stroke patients with aphasia; CG: control group; CG1: sub‐group of CG aged ≥55 years, which corresponds to age range of stroke patients.

** *p* < 0.01 according to Kruskal–Wallis test.

The CG consisted of 126 subjects (63 men and 63 women, with the median age of 54.5 years [range 18–89, QD = 35.25]). With respect to level of education, the subjects were distributed equally among basic, secondary, and high levels.

A sub‐group of the CG aged ≥55 years, which corresponds to age range of left hemisphere stroke patients (CG1), comprised 63 individuals, including 30 men and 33 women (median age 72, range 55–88 years, QD = 15.5), with an almost equal distribution among their levels of education.

### 2.2 Measures

The MASTest consists of nine subtests for assessing expressive (subtests 1–3 and 8–9: Naming, Automatic Speech, Repetition, Verbal Fluency and Written/Spelling to Dictation subtest) and receptive language (subtests 4–7: Yes/No Accuracy, Object Recognition from Field of Five, Following Verbal Instructions, Reading Instructions subtest).

Two points are given for each correct answer and zero points for each incorrect answer. The only exception is the Verbal Fluency subtest in which the scoring is different (0 points given for 0–5, 5 points for 5–10, and 10 points for 11 and more intelligible verbalizations). There are two possibilities for analyzing patients’ performance: (a) to write all words that the patient verbalizes and code unintelligible utterances with a dash; (b) to tape the patients’ response and transcribe it afterward. Each subtest adds up to 10 points, except for the fourth subtest (Yes/No Accuracy), which adds up to 20. The sum of subtests 1–3 and 8–9 scores forms the MAST‐E (range 0–50), while the subtests 4–7 form the MAST‐R (range 0–50), and the sum of all subtests forms the MAST‐T (range 0–100). The MAST‐T helps to determine the presence of aphasia and its severity. By comparing MAST‐E and MAST‐R, it is possible to get a primary impression of which language domain is more damaged: expressive or receptive language.

In the original version of MAST, there is a possibility to give optional ratings (presence or absence), such as dysarthria, paraphasia, perseveration, and orientation. These do not affect the MAST‐T. Stimulus materials include one photograph, five written instructions (each instruction on a separate page) and five common everyday objects (e.g., pen, keys, watch). In the subtests Object Recognition from Field of Five and Written/Spelling to Dictation, a table or hard folder is needed.

### 2.3 Procedure

The English version of the MAST was translated into the Estonian language and then back into English. After that, the versions (the original and the translation) were compared and adaptations were made with respect to language and cultural specifics. The changes made to the MASTest compared to the original MAST are listed in Table 2. No adaptations were made in the Naming, Object Recognition from Field of Five, Following Verbal Instructions, or Reading Instructions subtests.

**Table 2:** Adaptations of MASTest

Language domain	Subtest	Adaptions
Expressive language	Subtest 2 Automatic Speech	Items 1–2 no adaptions were made, items 3–5 Estonian proverbs and sayings.
	Subtest 3 Repetition	All items were adapted—original words were not translated, Estonian words were chosen based on original words’ phoneme and syllable structure.
	Subtest 8 Verbal Fluency	New photograph was chosen (“Christmas Eve”), taking Estonian cultural context into account.
	Subtest 9 Written/Spelling to Dictation	Original words were not translated, Estonian words were chosen based on original words’ phoneme and syllable structure.
Impressive language	Subtest 4 Yes/No Accuracy	Item 3 was adapted—Estonian location (island of Saaremaa) was chosen.

The adjusted MASTest was then applied by expert speech and language therapists to five people with and five people without aphasia to test the usability and interpretability of the translated version of the test. Based on the expert speech and language therapists’ judgments about the content of the tasks, the comprehensibility of the instructions, the procedure of administering the test and evaluating the results, and the test’s content validity were rated “good” and no further alterations were made.

The LHA+ group was assessed at the hospital. The CG was assessed at home or other places (such as a home for the elderly, a daycare center for the elderly, and army bases).

An evaluation was performed within 2–4 days of the onset of stroke. The MASTest was performed at the patients’ bedside. A letter chart was used in some cases where stroke patients were unable to use their right hand due to right side hemiparesis and refused to write with their left hand. In a verbal fluency subtest, the patients’ response was tape‐recorded and transcribed afterward. A rating form was filled out while administering the test. The Minimental State Examination (MMSE; Folstein, Folstein, & McHugh, 1975) was performed on all recruited healthy controls (controls <24 points were excluded).

The presence of aphasia was determined during a bedside clinical logopedic examination with a nonstandardized test which was performed by a qualified speech and language therapist. The MASTest (both in the LHA+ and CG) was performed by a qualified speech and language therapist and three previously instructed speech and language therapy students.

Information about patients’ education was obtained from the patients or their relatives. Patients’ level of consciousness and cooperability were assessed by a neurologist and a speech and language therapist by using the Glasgow Come Scale (GCS). Patients with GCS corresponding to degree >14 were included.

Demographic data (age) and medical information (neuroimaging findings, stroke onset, recurring stroke, and prestroke dementia diagnosis) were obtained from patients’ medical history. The study was approved by the Research Ethics Committee of the University of Tartu. Written informed consent was obtained from stroke patients or their relatives. Participants in the CG signed the consent form themselves.

### 2.4 Statistical analysis

The program SPSS (version 17.0) was used to analyze the data. Frequency calculation, mean values, dispersion, Pearson correlation coefficient, and bar charts were used to describe the subjects. Cronbach’s alpha (*α*) was used to evaluate the internal consistency of the MASTest. The results are considered acceptable *α* ≥ 0.7 (George & Mallery, 2003). The distribution of the MASTest‐T, MASTest‐E, and MASTest‐R scores was nonnormal both in the CG and in the LHA+ group (*p* < 0.01; χ^2^ test). As the distribution of data did not correspond to the normal distribution, nonparametric tests were applied. To compare the groups, the Mann–Whitney *U* test, the Kruskal–Wallis test, and χ^2^ tests were used. The result was considered statistically significant when the *p*‐value was <0.05. To assess the sensitivity and specificity of the MASTest‐R program, ROC analysis was used.

## 3 RESULTS

### 3.1 MASTest scores according to age and level of education

The MASTest summary statistics and median score values stratified by age, gender, and level of education are presented in Table 3. MASTest scores were significantly associated with both age (Mann–Whitney *U* test; MASTest‐T: *U* = 1,142.50, *Z* = 4,101, *p* = 0.000***; MASTest‐E: *U* = 1,353.00, *Z* = 3.079, *p* = 0.002**; MASTest‐R: *U* = 1,372.00, Z = 2.986, *p* = 0.002**) and level of education (Kruskal–Wallis test; MASTest‐T: *x* ^2^ = 13.640, *p* = 0.000***; MASTest‐E: *x* ^2^ = 8.923, *p* = 0.012**; MASTest‐R: *x* ^2^ = 10.241, *p* = 0.006**).

**Table 3:** MASTest scores in CG (*n* = 126) and proposed normal limits for the CG1 (*n* = 48)

MASTest parameter: possible score range	MASTest‐T (0–100)	MASTest‐E (0–50)	MASTest‐R (0–50)
Summary statistics: median score (range)	100 (71–100)	50 (33–50)	50 (32–50)
Age: median score (range) and quartile deviation (QD)	Age 18–54 (*n = *63): 100 (79–100), QD* = *4 CG1 (*n = *63): 95 (71–100)***, QD* = *8.5	Age 18–54: 50 (41–50), QD* = *0 CG1: 48 (33–50)**, QD* = *5	Age 18–54: 50 (36–50), QD* = *2 CG1: 48 (32–50)**, QD* = *6
Gender: median score (range) and quartile deviation (QD)	M (*n = *63): 96 (71–100), QD* = *8 W (*n = *63): 98 (73–100), QD* = *4.5	M: 50 (33–50), QD* = *5 W: 50 (40–50), QD* = *2	M: 50 (36–50), QD* = *4 W: 50 (32–50), QD* = *2
Level of education: median score (range) and quartile deviation (QD)	B (*n* = 42): 95.5 (71–100), QD* = *8.75 S (*n = *42): 100 (82–100), QD* = *4.75 A (*n* = 42): 100 (80–100)***, QD* = *5	B: 48 (33–50), QD* = *5 S: 50 (38–50), QD* = *2 A: 50 (44–50)*, QD* = *0	B: 48 (32–50), QD* = *5.5 S: 50 (40–50), QD* = *2 A: 50 (36–50)**, QD* = *2
Normal score limits for CG1: 5th percentile (stratified according to level of education)	B: 73 S: 83 A: 91	B: 34 S: 41 A: 45	B: 34 S: 42 A: 42

Note

CG: the healthy volunteers control group; CG1: a sub‐group of CG aged ≥55 years which corresponds to age range of left hemisphere stroke patients with aphasia.

MASTest‐T: total score; MASTest‐E: expressive score; MASTest‐R: receptive score.

M: men; W: women; B: basic education; S: secondary education; A: academic education.

^*^ *p* < 0.05; ^**^ *p < *0.01; ^***^ *p < *0.001 according to Mann–Whitney *U* test (age and gender) and Kruskal–Wallis test (level of education).

Proposed normative values for CG1 stratified by level of education are shown in Table 3. Within CG1, no significant correlation between MASTest scores and age was recorded (MASTest‐T: *r* = −0.150, *p* > 0.05).

### 3.2 Comparison of the values of MASTest scores between LHA+ group and CG

MASTest scores and subtest values were significantly different (*p* < 0.001) in the LHA+ group and CG (Table 4).

**Table 4:** MASTest median values of TI, RI, EI, and *p*‐values in the LHA+ group and CG

Score Subtest (maximum points)	Sample	Group median	Range (limits)	Quartile deviation (QD)	*Z*	*p*
*MASTest‐E (50)*	LHA+ group	32	48 (0–48)	25	−9.676	<0.001
	CG	50	17 (33–50)	5		
Naming (10)	LHA+ group	8	10 (0–10)	5.5	−8.870	<0.001
	CG	10	2 (8–10)	0		
Automatic Speech (10)	LHA+ group	10	10 (0–10)	4	−5.240	<0.001
	CG	10	4 (6–10)	0		
Repetition (10)	LHA+ group	8	10 (0–10)	4	−6.982	<0.001
	CG	10	4 (6–10)	0		
Verbal Fluency (10)	LHA+ group	5	10 (0–10)	5	−8.680	<0.001
	CG	10	10 (0–10)	0		
Written/Spelling to Dictation (10)	LHA+ group	2	10 (0–10)	8	−9.823	<0.001
	CG	10	10 (0–10)	0		
*MASTest‐R (50)*	LHA+ group	40	38 (12–50)	16	−7.191	<0.001
	CG	50	18 (32–50)	4		
Yes/No Accuracy (20)	LHA+ group	18	18 (2–20)	6	−4.090	<0.001
	CG	20	12 (8–20)	1.5		
Object Recognition from Field of Five (10)	LHA+ group	10	10 (0–10)	2	−7.076	<0.001
	CG	10	0 (10–10)	0		
Following Verbal Instructions (10)	LHA+ group	8	10 (0–10)	6	−7.728	<0.001
	CG	10	6 (4–10)	0		
Reading Instructions (10)	LHA+ group	6	10 (0–10)	6	−7.508	<0.001
	CG	10	6 (4–10)	2		
*MASTest‐T (100)*	LHA+ group	73	84 (14–98)	30.75	−8.825	<0.001
	CG	98	29 (71–100)	7		

Note

LHA+ group: left hemisphere stroke patients with aphasia, *n = *50; CG: control group, *n = *126.

The MASTest‐T in the LHA+ group varied between 14 and 98, which means that there was no ceiling or floor effect in respect to the MASTest‐T. In subtests and MASTest‐R, some patients of the LHA+ group achieved the maximum values. However, the ceiling effect was obvious in the CG (57 out of 126 persons scored 100 points on the MASTest‐T), and in the Object Recognition from Field of Five subtest all 126 persons scored 10.

### 3.3 Validity of MASTest based on diagnostic accuracy

The sensitivity and specificity of MASTest were first evaluated using the 5th percentiles of the values in the CG (Kostalova et al., 2008) using empirical cutoff values (82.5 for MAST‐T, 41.5 for MASTest‐E, 40 for MASTest‐R).

The sensitivity of the MASTest (correct detection of abnormal MASTest scores in the LHA+ group) was 74% for the MASTest‐T and the MASTest‐E (13 out of 50 in the LHA+ group had a normal MASTest‐T or MASTest‐E), and 64% for the MASTest‐R (18 out of 50 patients had a normal MASTest‐R).

Similarly, the specificity of the MASTest (correct detection of MASTest scores in the CG) was 94% for the MASTest‐TI and MASTest‐EI (119 out of 126 patients) and 95% (120 out of 126) for the MASTest‐RI.

### 3.4 ROC analysis of the MASTest scores

**Figure 1:**
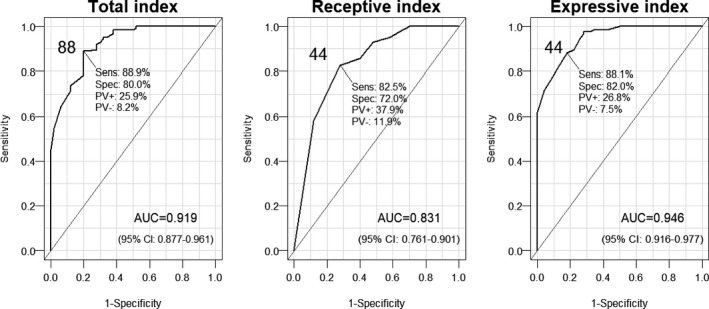
Fitted receiver operating characteristic curves for CG. Cutoff values in solid numbers, Sens and Spec: sensitivity and specificity; PV+ and PV−: positive and negative predictive values. AUC (area under curve) with 95% confidence limits is also shown. *N = *126 (all education levels included)

The sensitivity and specificity of MASTest scores in detecting aphasia were also assessed with ROC analysis, which yielded higher cutoff values than the empirical cutoff values in the previous test (88 for MASTest‐T, 44 for MASTest‐E and MASTest‐R). All fitted curves revealed statistically significant AUC (“area under curve”; Figure 1). The MASTest‐T as well as the MASTest‐E, and to a lesser extent, the MASTest‐R provided sufficiently high sensitivity and specificity for diagnostic differentiation between the LHA+ group and the CG.

### 3.5 Internal consistency of MASTest

The internal reliability of the MASTest‐T, MASTest‐E, and MASTest‐R, assessed using Cronbach’s alpha, was good both in the LHA+ group and in the CG (0.885–0.947). Acceptable results with consistent responses were obtained with the CG MASTest‐T and MASTest‐R (0.730–0.776). However, with the CG MASTest‐E, values were inconsistent (0.400; Table 5).

**Table 5:** Internal consistency (Cronbach's alpha) of MASTest

Score	LHA+ group, *n = *50	CG, *n = *126	LHA+ group and CG, *n* = 176
MASTest‐E	0.885	0.400	0.905
MASTest‐R	0.936	0.776	0.911
MASTest‐T	0.940	0.730	0.947

Note

LHA+ group: left hemisphere stroke patients with aphasia, *n* = 50; CG: control group, *n* = 126.

## 4 DISCUSSION

In this study, we presented our process of adapting and validating the MASTest for Estonian‐speaking persons. The process of translating and adapting the MASTest was in line with the translational versions of the MAST (Khatoonabadi et al., 2015; Kostalova et al., 2008; Nagendar & Ravindra, 2012; Nakase‐Thompson et al., 2005, 2002; Romero et al., 2012). The equivalency of the MASTest with the original English version (Nakase‐Thompson et al., 2005) ensures that the results of the MASTest are comparable to the results of the MAST in English as well as other languages. The standard methodology used in developing the MASTest ensured the face and content validity of the MASTest. Our results further demonstrated that the MASTest has a high discriminative validity and a high internal consistency for the whole sample and the LHA+ group.

This study on the MASTest was performed on the LHA+ group in the acute phase of stroke (on the 2nd to 4th day of hospitalization). In other studies (including the original study by Nakase‐Thompson et al.), patients were screened at a later time, in the subacute or chronic phase of stroke (Khatoonabadi et al., 2015; Kostalova et al., 2008; Nagendar & Ravindra, 2012; Nakase‐Thompson et al., 2005, 2002; Romero et al., 2012). Our results suggest that the MAST can also be effectively used in the acute phase of stroke, which is important for the early diagnosis of aphasia and to determine the localization and extent of brain injury, which can maximize the benefits of therapy. Screening tests give information on the severity of language disorder in the fields of language production, comprehension, and oral and written language (Pedersen, Vinter, & Olsen, 2004; Vogel, Maruff, & Morgan, 2010). Based on the data collected during the short period of hospitalization (during the acute phase), decisions for further treatment and rehabilitation can be made (Inatomi et al., 2008).

MASTest scores in the CG were associated with age and level of education. Younger and more educated healthy individuals achieved higher MASTest scores, which is in accordance with other studies (Nagendar & Ravindra, 2012; Romero et al., 2012). We did not reveal a significant correlation between MASTest scores and age within CG1 (individuals corresponding by age to the LHA+ group). Therefore, normative score limits for CG1 were stratified by level of education (Table 3). However, stroke with aphasia can also be diagnosed in persons <55 years old (Singhal et al., 2013). In these rare cases, higher normal score limits should be considered.

Median MASTest scores across the three summary scores (MASTest‐T, MASTest‐E, MASTest‐R) and the nine subtests were all significantly different in the LHA+ group and CG, demonstrating the high sensitivity and specificity of MASTest in detecting language impairment. This was also demonstrated in the original (Nakase‐Thompson et al., 2005) and translated tests (Khatoonabadi et al., 2015; Nagendar & Ravindra, 2012).

The ceiling and floor effects of the MAST have been studied in respect to the MASTest‐T of the LHA+ group and CG. In our study, no patients scored 0 or 100 on the MASTest‐T (limits 14 and 98). The lack of floor or ceiling effects further verifies the content validity of the MASTest. The floor or ceiling effects were also not reported for the original English (Nakase‐Thompson et al., 2005) and some translated versions (Khatoonabadi et al., 2015; Nagendar & Ravindra, 2012; Romero et al., 2012). However, a ceiling effect was reported in some subtests within the LHA+ group and it is common in the CG (Khatoonabadi et al., 2015), which indicates that the MASTest is easy to perform for many of the healthy persons in the CG. This probably also affected the sensitivity and specificity of the MASTest.

The sensitivity and specificity of the MASTest were assessed using both the 5th percentiles of the values in the CG and ROC analysis. The specificity of the MASTest evaluated using 5th percentiles of the MASTest‐T was good (94%), but sensitivity was lower (74%), which is lower than in some other studies (Kostalova et al., 2008; Romero et al., 2012). In the original version of the MAST (Nakase‐Thompson et al., 2005) 62%–76% (depending on which subtests or summary scores were used) of the LHA+ group, members were correctly classified. In our study, aphasia was not detected by the MASTest in 26% persons in the LHA+ group. However, 6% of persons in the CG had MASTest‐T results below the cutoff point (82.5%). This might have been due to the fact that some elderly individuals in the CG had mild cognitive impairment which was not detected by the MMSE. ROC analysis yielded higher cutoff values (88% for the MAST‐T) and sensitivity (88.9%), but lower specificity (80.0%) values than the first method. A smaller proportion of the LHA+ group (11.1%) and a higher proportion of the CG (20.0%) are not correctly classified. This means that more persons in the CG have to be thoroughly tested for the presence of aphasia.

In both cases (5th percentiles and ROC analysis), the sensitivity and specificity were lower for the MASTest‐R than for the MASTest‐E and MASTest‐T. In two other studies where ROC analysis was performed (Kostalova et al., 2008; Romero et al., 2012), MAST‐R sensitivity and specificity were also lower than in the case of the MAST‐T and MAST‐E. It seems that language comprehension tasks are harder to correctly accomplish than language production tasks for the CG.

The internal reliability of the MASTest (Cronbach’s alpha) was good for the whole sample and the LHA+ group, acceptable for the CG for the MASTest‐T and MASTest‐R, and low for the CG for the MASTest‐E. The low internal consistency of the MASTest‐E in the CG probably indicates the low variability of indices of language production with assessing tasks in this group.

In our study, we were unable to assess the convergent validity of the MASTest as no other screening or comprehensive test batteries for aphasic patients are available in Estonian. Also, intra‐observer validity and test–retest reliability were not assessed. Our CG consisted only of healthy individuals, and patients with right‐hemisphere stroke were neglected as their performance in the MAST has been described in earlier studies (Nakase‐Thompson et al., 2005; Romero et al., 2012). In addition, our LHA+ group consisted of only a few individuals with a high level of education.

Our study was performed during the acute phase of stroke; all earlier studies, including the original study (Nakase‐Thompson et al., 2005), were conducted during the subacute or chronic phase of stroke. Early detection of aphasia will enable early rehabilitation and thereby improve language recovery outcomes.

The MASTest is the first validated aphasia screening test for Estonian‐speaking people, who number less than one million worldwide. Our experience indicates that the MAST can be used for small nations, but getting a comprehensive sample of the LHA+ group for test validation may be complicated. Simultaneously, collecting data in several hospitals and rehabilitation centers may be a useful strategy to shorten the period of validation.

The MASTest was developed as a brief screening tool that could be administered at the bedside or during clinic appointments by a variety of healthcare providers. Medical personnel are often asked to comment on patients’ cognitive abilities, including language function and communication skills, which has implications for implementing medical and rehabilitation interventions and monitoring the course of recovery (Nakase‐Thompson et al., 2005). The original English version as well as the Estonian and other translated versions have proved to be valid and reliable instruments to assess language disorder in patients with post‐stroke aphasia. To facilitate its practical use for medical personnel, complementation of the test manual, which the author is planning to compile, is needed.

## CONFLICT OF INTEREST

This study was supported by the Grants PUT1239 and IUT2‐4 of the Estonian Research Council.
